# CpxR negatively regulates the production of xenocoumacin 1, a dihydroisocoumarin derivative produced by *Xenorhabdus nematophila*

**DOI:** 10.1002/mbo3.674

**Published:** 2018-06-11

**Authors:** Shujing Zhang, Xiangling Fang, Qian Tang, Jing Ge, Yonghong Wang, Xing Zhang

**Affiliations:** ^1^ Research and Development Center of Biorational Pesticides Key Laboratory of Plant Protection Resources and Pest Management of Ministry of Education Northwest A &F University Yangling Shaanxi China; ^2^ State Key Laboratory of Grassland Agro‐ecosystems College of Pastoral Agriculture Science and Technology Lanzhou University Lanzhou China; ^3^ School of Agriculture and Environment Faculty of Science The University of Western Australia Crawley Western Australia Australia

**Keywords:** antimicrobial activity, biosynthesis regulation, CpxR, *X*. *nematophila*, xenocoumacin 1

## Abstract

Xenocoumacin 1 (Xcn1), a major antimicrobial compound produced by *Xenorhabdus nematophila*, has great potential for use in agricultural productions. In this study, we evaluated the effects of CpxR, a global response regulator associated with the mutualism and pathogenesis of *X*. *nematophila*, on the antimicrobial activity and Xcn1 production. The mutation of *cpxR* could promote the production of Xcn1 significantly with its level in *ΔcpxR* mutant being 3.07 times higher than that in the wild type. Additionally, the expression levels of *xcnA‐L* genes, which are responsible for the production of Xcn1, were increased in *ΔcpxR* mutant while the expression levels of *xcnMN*, which are required for the conversion of Xcn1 into Xcn2 was reduced. Noticeably, Xcn2 was also enhanced on account of the conversion of excessive Xcn1 in spite of low expression levels of *xcnM* and *xcnN* in *ΔcpxR* mutant. The transcriptional levels of *ompR* and *lrp*, encoding the global response regulators OmpR and Lrp which negatively and positively regulate the production of Xcn1 were concurrently decreased and increased, respectively. Correspondingly, *ΔcpxR* mutant also exhibited increased antimicrobial activities in vitro and in vivo. Together, these findings suggest that CpxR negatively regulates *xcnA‐L* genes expression while positively regulating *xcnMN* expression in *X*. *nematophila* YL001, which led to a high yield of Xcn1 in *ΔcpxR* mutant.

## 1 INTRODUCTION

*Xenorhabdus nematophila*, a mutualistic symbiont of the soil‐dwelling nematode *Steinernema carpocapsae*, is a potent producer of natural bioactive compounds. Whole‐genome sequencing programs have revealed that *X. nematophila* had great biosynthetic potential in secondary metabolites. In *X. nematophila* ATCC 19061 7.5% of the genomic genes encode the proteins involving in secondary metabolism. The majority of these encoded molecules, however, are cryptic (Bisch et al., 2016; Chaston et al., 2011). Until now, *X. nematophila* has been known to produce several secondary metabolites with antimicrobial activity, including xenocoumacins (Xcns) (Huang et al., 2005; Lang, Kalvelage, Peters, Wiese, & Imhoff, 2008; Yang et al., 2011; Zhou, Yang, Qiu, & Zeng, 2017), indole derivatives (Li, Chen, & Webster, 1997; Li, Chen, Webster, & Czyzewska, 1995; Sundar & Chang, 1993), peptides (Boszormenyi et al., 2009; Fuchs, Proschak, Jaskolla, Karas, & Bode, 2011; Gualtieri, Aumelas, & Thaler, 2009), benzylineacetone (Ji et al., 2004), and nematophin (Li et al., 1997). These metabolites not only have diverse chemical structures but also a wide range of bioactivities of medicinal and agricultural interests.

Xenocoumacins, including Xcn1 and Xcn2, are the major antimicrobial compounds produced by *X. nematophila*. Xcn1 exhibits a broad antimicrobial activity against Gram‐positive bacteria and a strong antifungal activity (*Alternaria alternata*,* Botrytis cinerea*,* Rhizoctonia solani*, and *Phytophthora* species) (Huang, Yang, & Yang, 2006; Huang et al., 2005; Zhou et al., 2017). Xcn2, however, shows substantially reduced bioactivities (Mcinerney, Taylor, Lacey, Akhurst, & Gregson, 1991; Yang et al., 2011; Zhou et al., 2017). It has been proved that 14 genes (*xcnA*‐*xcnN*) involved in Xcn synthesis in *X. nematophila*. The genes (*xcnA‐L*) are responsible for Xcn1 synthesis and the conversion of Xcn1 into Xcn2 is controlled by *xcnM* and *xcnN* that encode proteins homologous to saccharopine dehydrogenases and fatty acid desaturases, respectively (Park et al., 2009). When Xcn2 production is attenuated, an increase in Xcn1 is observed, along with a 20‐fold reduction in cell viability, suggesting that the conversion of Xcn1 into Xcn2 is a resistance mechanism utilized by the bacteria to avoid self‐toxicity (Park et al., 2009). Although Xcn1 has great potential for using as a new biopesticide in agricultural productions, its low yield in *X. nematophila* wild strains is a substantial limitation for its practical applications.

*X. nematophila* can adapt to the changing environmental conditions to modulate the pathogenic and mutualistic behaviors to its host, which is closely associated with the production of bioactive substances (Herbert, Cowles, & Goodrich‐Blair, 2007; Herbert & Goodrich‐Blair, 2007). Many previous researches have confirmed that the antimicrobial activity of *X. nematophila* varies according to the fermentation conditions (Cowles, Cowles, Richards, Martens, & Goodrich‐Blair, 2007; Furusawa et al., 2008; Goodrich‐Blair, 2007; Wang, Fang, An, Wang, & Zhang, 2011; Wang, Fang, Li, & Zhang, 2010; Wang, Li, Zhang, & Zhang, 2008). But it still remains unclear how *X. nematophila* recognizes these changes or how these signals are associated with the antimicrobial activity. Lrp (leucine‐responsive protein), a global regulator of transcription, serves as a sensor of intracellular metabolic status and thus generally associates with the response to nutrient availability (Brinkman, Ettema, De Vos, & Van Der Oost, 2003; Hart & Blumenthal, 2011). In *X. nematophila*, as a global regulator as well, Lrp can regulate the pathogenic and mutualistic interactions, especially affect the production of secondary metabolites (Cowles et al., 2007; Goodrich‐Blair, 2007; Hussa, Casanovatorres, & Goodrichblair, 2015). Lrp is predominantly a positive regulator of secondary metabolite production. The mutation of *lrp* could significantly reduce the production of antibiotics, including xenortides, xenematides, and Xcn1. Correspondingly, its mutant exhibited no antimicrobial activities against *Micrococcus luteus* and *Bacillus subtilis* (Cowles et al., 2007; Engel, Windhorst, Lu, Goodrichblair, & Bode, 2017; Goodrich‐Blair, 2007). Both two‐component systems, CpxRA and EnvZ/OmpR, are involved in responding to the nematode and other insect environments, which regulate the mutualistic and pathogenic interactions of *X. nematophila* with its host (Herbert & Goodrich‐Blair, 2009; Herbert et al., 2007; Park & Forst, 2006). As a sensor histidine kinase, CpxA and EnvZ can sense diverse signals including the changes in pH and osmolarity. Upon recognition of these signals, CpxA and EnvZ autophosphorylate and then donate their phosphoryl groups to CpxR and OmpR, respectively. After being phosphorylated, the cognate response regulator can bind to a specific promoter sequence of a target gene and subsequently regulate its expression (Jubelin et al., 2005). The increased osmotic stress has been found to generally stimulate metabolite production of *X. nematophila* (Crawford, Kontnik, & Clardy, 2010). As EnvZ senses high osmolarity of hemolymph, EnvZ phosphorylates OmpR to activate this response regulator (Forst & Boylan, 2002). It was shown that OmpR repressed flagella synthesis, exoenzyme, and antibiotic production of *X. nematophila* by negatively regulating the *flhDC* operon (Park & Forst, 2006). Moreover, OmpR also negatively regulates the expression of *xcnA‐L* and positively regulates the expression of *xcnMN* in *X. nematophila* (Park et al., 2009).

As a response regulator of great importance, CpxR is also involved in regulating the mutualism and pathogenesis of *X. nematophila*. It can positively influence the motility, secreted lipase activity, and transcription of *lrhA* necessary for the virulence as well as the expression of *nil* genes responsible for mutualistic colonization of nematodes (Herbert & Goodrich‐Blair, 2009). In addition, CpxR negatively influences the production of antibiotic activities, protease, and secreted hemolysin. The *ΔcpxR*1 mutant strain exhibited increased antimicrobial activity against *B. subtilis*, while the antimicrobial activity of complementary strain *ΔcpxR1* (Tn7/*cpxRA*) was restored to the comparative level of the wild type (Herbert et al., 2007). However, to our best knowledge, there is no report about the regulatory effects of CpxR on the Xcn1 production and the antimicrobial activity of *X. nematophila* against phytopathogens. Therefore, in this study, we constructed a *ΔcpxR* mutant of *X. nematophila* YL001 to determine the influences of CpxR on the Xcn biosynthesis and the antifungal activities of *X. nematophila* against *Botrytis cirerea*, in vivo and in vitro.

## 2 EXPERIMENTAL PROCEDURES

### 2.1 Bacterial strains and growth conditions

*X. nematophila* YL001 was isolated from its nematode symbiont, *Steinernema* sp. YL001 obtained from the soil from Yangling, China, and had been identified according to its morphological and molecular characteristics.

Details of the strains and plasmids used in this study were provided in Table 1. *X. nematophila* strain was grown in TSB medium (tryptic soy broth) at 28°C. *Escherichia coli* was grown at 37°C either in Luria‐Bertani medium (LB: 1.0% Bacto tryptone, 0.5% yeast extract, and 1% NaCl) by shaking at 180 rpm or on corresponding solid agar media (1.5% agar) as needed.

**Table 1:** Bacterial strains and plasmids used in this study^a^

Strains	Relevant genotype, phenotype, or characteristic (s)	Source
*X. nematophila*		
YL001	Wild‐type, phase I variant; Amp^r^	Laboratory stock
*ΔcpxR*	YL001*ΔcpxR*::Km^r^	This study
*E. coli*		
DH5α (λpir)	General cloning strain	TAKARA
S17‐1(λpir)	*recA*,* thi*,* pro*,* hsd*R‐M + . RP4‐2Tc::Mu Km::Tn7 in the chromosome; donor strain for conjugations	Laboratory stock
Plasmids		
pDM4	Suicide vector; Cm^r^, Suc^s^, *ori*R6K, *mob*RP4	Laboratory stock
pDM4*cpxR*Km^r^	Plasmid pDM4 carrying 1,105‐bp *cpxR* 5′ upstream region, 973‐bp kanamycin‐resistant cassette, and 1,192‐bp *cpxR* 3′ downstream region	This study
pMD19T	Cloning vector; Amp^r^	TAKARA
pMD19T*cpxR*Km^r^	pMD19T carrying 1,105‐bp *cpxR* 5′ upstream region, 973‐bp kanamycin‐resistant cassette, and 1,192‐bp *cpxR* 3′ downstream region	This study
pJCV53	Source of Km^r^ gene	Laboratory stock

a Amp^r^, Ampicillin resistance; Km^r^, Kanamycin resistance; Cm^r^, Chloramphenicol resistance.

Antibiotics were used when needed at the following concentrations: ampicillin (150 μg/ml for *X. nematophila* and 50 μg/ml for *E. coli*), chloramphenicol (25 μg/ml for both *X. nematophila* and *E. coli*), and kanamycin (50 μg/ml for both *X. nematophila* and *E. coli*).

### 2.2 DNA manipulation

DNA and plasmid isolation, restriction digests, PCR, ligation reactions, and gel electrophoresis were conducted according to the standard protocols of molecular Cloning. PCR amplification was conducted using Ex Taq (Takara Otsu, Shiga, Japan) according to the manufacturer’s directions. Prime STAR^®^ Max was used as the DNA Polymerase (Takara Otsu, Japan). PCR‐amplified fragments were recovered from agarose gels using the Mini‐Best DNA Fragment Purification Kit (Takara Otsu, Japan). Primers for PCR amplification were designed using Primer Premier 5.0 software (Table S1). Recombinant constructs were verified by DNA sequencing.

### 2.3 Construction of *cpxR* mutant strain

Fragments, carrying upstream (1105‐bp) and downstream (1192‐bp) of *cpxR*, were amplified with primer pairs *cpxR*‐up‐F/*cpxR*‐up‐R and *cpxR*‐down‐F/*cpxR*‐down‐R, which contained engineered restriction enzyme sites, from YL001 chromosomal DNA. The PCR fragment containing kanamycin‐resistant cassette (973‐bp) was amplified using compatible restriction sites with primer pairs *Km*‐F/*Km*‐R from pJCV53. Fused PCR was performed in an additional amplification step via complementary DNA regions and its product was cloned into a pMD19T via the SphI and SacI restriction sites to create a pMD19TcpxRKm^r^. The fused fragment of upstream and downstream of *cpxR* and Km^r^ was PCR screened from pMD19T*cpxR*Km^r^ and cloned into the suicide vector pDM4 using SacI and SphI sites, creating pDM4*cpxR*Km^r^. The resulted plasmids were transformed into *E*. *coli* S17‐λ*pir* and conjugally transferred into the wild‐type strain of *X. nematophila* YL001. The mutant strain was identified based on the described method (Park et al., 2009).

### 2.4 Measurement of the growth rate

YL001 and *ΔcpxR* cells were grown overnight in TSB medium at 28°C by shaking at 180 rpm. Then, the cells were resuspended in TSB medium with an initial OD_600_ value of 0.01, respectively. Under the identical conditions, the growth rates were monitored every 6 hr during the 72‐hr period using a UV‐3310 spectrophotometer (Hitachi, Japan). Before testing, each sample solution was fully dispersed using a moderate ultrasonic amplitude (50%) for 5 min to avoid the probable cell aggregation. Each experiment was performed in triplicate.

### 2.5 Cell‐free filtrate and methanol extract preparation

Nine percent (v/v) of the seed culture was used as the inoculum. Cultures of the wild type and *ΔcpxR* mutant of *X. nematophila* YL001 were conducted in 250 ml flask containing 100 ml TSB medium (Wang et al., 2010, 2011). Each flask was incubated on a rotary shaker at 28°C and 180 rpm for 72 hr and then centrifuged (12,000*g*, 20 min, 4°C) to separate cells. The filtrate was collected and stored at 4°C until use. The methanol extract of the cell‐free culture was prepared according to previous methods (Boszormenyi et al., 2009; Fang, Li, Wang, & Zhang, 2011; Fang, Zhang, Tang, Wang, & Zhang, 2014). Briefly, cell‐free filtrates (500 ml) of the *ΔcpxR* mutant and the wild type were mixed, respectively, with activated D101 polymeric adsorbent resin at 1:20 (v/v) and incubated for 24 hr. The resin was separated by a G3 glass filter, washed with distilled water, and 25% methanol consecutively, and then placed on the top of the column filled with activated D101. After washing the column with distilled water, methanol was pumped onto the column at 10 ml/min and the eluate was collected in 200 ml aliquots. The methanol extract was dried at 40°C and stored at 4°C until use.

### 2.6 Analysis of the production of Xcns in the *ΔcpxR* mutant and the wild‐type strain

The analysis of the production of Xcns was performed according to the reported method (Engel et al., 2017; Park et al., 2009) by a liquid chromatography‐tandem mass spectrometry (LC–MS/MS, API 2000, AB Sciex, USA). The equivalent weight of dried methanol extract (8 mg) of the wild‐type and mutant strain was resuspended in 8.0 ml methanol for analysis. HPLC separation was performed by using an Agilent C_18_ column (150 mm × 4.6 mm, 5 μm) and a water/acetonitrile gradient (+0.1% formic acid) (gradient: 0–14 min, 5%–95% acetonitrile, injection volume: 5 μl). A full scan mode (*m*/*z* 100–1,000) was applied for MS. For quantification of Xcn1 and Xcn2, the ions *m*/*z* [M+H]^+^ 466.3 and 407.3 were quantified, respectively. The relative amount of Xcn was calculated by the following equation: the relative amount of Xcn = the peak area of extracted ion chromatogram (EIC) of Xcn in *ΔcpxR* mutant/the peak area of extracted ion chromatogram (EIC) of Xcn in the wild type, the peak area was calibrated by its OD_600_ value, and the relative amount of Xcn of the wild type was referred to 1. All analyses were performed in triplicate.

### 2.7 Assay of antimicrobial activity

The antimicrobial activities of the cell‐free filtrate of the wild‐type and the *ΔcpxR* strain against five bacteria species (Table S2) were determined using an agar diffusion plate assay (Ji et al., 2004). The cell‐free filtrate samples were sterilized by filtration (0.22 μm) before use. Molten sterile NA medium (100 ml) in a flask was inoculated with a bacterial suspension (1.5 ml, 10^7^–10^8^ cfu/ml) at 45°C. Then, the mixture was poured into six sterile 9‐cm Petri dishes (15 ml each Petri dish) to form uniform plates. Sixty μl of the sterile samples was pipetted on sterile filter paper disks (Whatman No.1, 6 mm in diameter), which were allowed to dry in the sterile air. The dry disks were arranged on the inoculated plates for diffusion (each plate with three disks of a sample). The plates with paper disks were incubated at 28°C for 48 hr to determine the sizes of the inhibition zones. Paper disks impregnated with TSB medium were used as controls. Each experiment was repeated three times under the same conditions.

The inhibitory effects of the cell‐free filtrates of the wild‐type and *ΔcpxR* strain on different oomycete and fungal pathogens (Table 4) were determined according to the previously described methods (Fang et al., 2014). The pathogens were obtained from the Agricultural Culture Collection Institute, Northwest A & F University, China. Briefly, 1 ml of the cell‐free filtrate was mixed with 9‐ml potato dextrose agar (PDA) at 40°C and then the obtained mixture was poured into a 9‐cm Petri dish to form a PDA plate. One mycelial disk (0.4 × 0.4 cm) from the edge of 4‐day colony of a pathogen (Table 4) was put onto the PDA plate. There were three independent replications (three plates per replicate) for each experiment. PDA plates with fermentation medium were used as controls. The plates were incubated at 25°C under dark. After 7 days, the colony diameter of each plate was measured in two perpendicular directions and the inhibitory rate of the mycelial growth was determined according to the following formula: [(average colony diameter of control‐average colony diameter of treatment)/(average colony diameter of control − 4 mm)] × 100.

In vivo efficiencies of the methanol extracts of the wild‐type and *ΔcpxR* strain against *B. cinerea* were determined on tomato fruits according to the previously described methods (Fang et al., 2014). To determine the therapeutic effect, three tomato fruits with the similar size were placed at the bottom of a closed plastic container with moisture filter papers at the bottom to maintain high humidity. One mycelia agar disk (4 mm diameter) from the edge of 4‐day colony of *B. cinerea* was placed in the middle side of each fruit with mycelia side facing the surface of the fruit. The containers were placed in a climate chamber at 25°C. After 24 hr, the fruits were immediately sprayed with the methanol extract (1,000, 500, and 250 μg/ml) or the cell‐free filtrate. There were three replications (three fruits per replication) for each treatment. The controls for comparison were sprayed with water or 1,000 times dilution of the 50% carbendazim (Bianjing Plant Protection Technology Co., Ltd, Suzhou, China). To determine the protective effect, three tomato fruits were sprayed with each solution and kept under the same conditions as above. After 24 hr, the fruits were inoculated with *B. cinerea* as described above. After 7 days, lesion diameter of each fruit was measured in two perpendicular directions. The efficiency rate was determined by the formula as above.

### 2.8 Reverse transcription PCR (RT‐PCR) and quantitative real‐time PCR (qRT‐PCR) analysis

The *ΔcpxR* and wild‐type strains were cultured in 50‐ml fresh TSB media in a 250‐ml flask until to the logarithmic growth phase. The cells were harvested after incubation for 48 hr and their concentrations were dilute to OD_600_ of 0.6. Total RNA was extracted using SV Total RNA Isolation System (Promega) and the concentration was determined by optical density at 260 nm. The total RNA was treated with RNase‐free DNase I (Promega) to eliminate genomic DNA contamination before the reverse transcription. The control PCR reaction was conducted to examine if there was DNA contamination before RT‐PCR analysis. The quality of cDNA samples synthesized from 3‐μg DNase‐treated RNA was evaluated by the spectrophotometric method and agarose gel electrophoresis.

The primer sequences and accession numbers for all target and reference genes were provided in Table S1. qRT‐PCR was conducted using a SYBR Premix ExTaq II kit (TaKaRa, Dalian) on a thermo cycler CFX96 real‐time PCR detection system (Bio‐Rad, USA). cDNA served as the template for qRT‐PCR. The qRT‐PCR reactions were performed using 1 × SYBR Premix ExTaqTM, 0.4 μmol L^−1^ of each primer, and 2.5 μl RT reaction solutions in a final volume of 25 μl in triplicate. All qRT‐PCRs were performed in three technical replicates. The *recA* gene was used as the reference gene according to previous research (Park & Forst, 2006). The fold changes in the amount of *xcnA*,* xcnM*, and *xcnN* (target gene) transcript expression relative to the *recA* transcript (control gene) were determined using the previously described methods (Livak & Schmittgen, 2001; Park et al., 2009). The means of *Δ*(*Δ*Ct) and fold changes were calculated from three independent RNA samples.

### 2.9 Data analyses

All data analyses were performed using the SPSS statistical package (version 18.0 for windows; SPSS Inc., the USA). Subsequent multiple comparisons between treatments were evaluated according to the least significant differences (l.s.d) at *p *<* *0.05.

## 3 RESULTS

### 3.1 Construction of *ΔcpxR* strain

The DNA sequence lengths of downstream, Km, and upstream flanking regions were 1,198 bp, 974 bp, and 1,111 bp, respectively. The fusion PCR product of three parts was 3,283 bp in length (Supporting Information Figure S1). The confirmation of *ΔcpxR* mutant was performed with internal primers and the results showed that the DNA amplified from the wild type (693 bp) was in the expected position (between 500 and 750 bp) and the mutant strain did not have the gene *cpxR* (Supporting Information Figure S2). The PCR products of external primers were 1,202 bp long for the wild type and 1,040 bp long for the mutant strain (Supporting Information Figure S2). The PCR product of the mutant was sequenced. As shown in Supporting Information Figure S3, the upstream and downstream DNA fragments were complete and the gene *cpxR* in the *X. nematophila* was replaced with the screening tag, indicating that the gene *cpxR* was knocked out from *X. nematophila* YL001 successfully. The mutant was named as *ΔcpxR*.

### 3.2 Effect of CpxR on the growth pattern

In contrast to the wild type, *ΔcpxR* mutant displayed a slightly prolonged logarithmic phase, and the cell densities were lower at early and mid stages (Supporting Information Figure S4). These findings were consistent with the previous report, in which deleting *cpxR* resulted in taking longer to begin logarithmic growth (Herbert et al., 2007). At the late stage of fermentation, the cell density of *ΔcpxR* was higher than that of the wild type, whereas in previous study, no significant difference was observed between them (Herbert et al., 2007). This discrepancy may attribute to differences in growth conditions (LB vs. TSB) or strain identities (ATCC19061 vs. YL001). Besides, the growth pattern variations between the wild type and the *ΔcpxR* mutant may be associated with the ability to adapt rapidly to changing environmental conditions. CpxRA could sense the changing environment, and regulate the growth and metabolisms to adapt the environment of insect hemolymph (Herbert et al., 2007; Herbert & Goodrich‐Blair, 2009), deficiency of which may make it difficult to adapt its metabolism quickly to the nutrients available in hemolymph or various mediums.

### 3.3 Effect of CpxR on the production of Xcns

Previous study showed that deletion of *cpxR* in *X. nematophila* affected the production of antibiotics (Herbert et al., 2007). Thus, we investigated the variations in Xcns, the major antimicrobial compounds produced by *X. nematophila*, in *ΔcpxR* strain and the wild type by HPLC‐MS analysis. The results showed that the levels of Xcn1 and Xcn2 in *ΔcpxR* were 3.07‐ and 10.05‐fold higher than those in the wild‐type strain, respectively (Table 2, Supporting Information Figure S5).

**Table 2:** The relative amount of xenocoumacins (Xcn1 and Xcn2) in the wild type and the *ΔcpxR* mutant

Strain	Relative amount of Xcns^a^ (Arbitrary units OD^−1^)	
	Xcn1	Xcn2
Wild type	1	1
*ΔcpxR*	3.07 ± 0.52*	10.05 ± 1.97*

a The peak area of extracted ion chromatogram (EIC) of Xcn in *ΔcpxR* mutant/the peak area of extracted ion chromatogram (EIC) of Xcn in the wild type, the peak area was calibrated by its OD_600_ value, and the relative amount of Xcn of the wild type was referred to 1. Data are presented as the average ± *SD* for three replicates. An asterisk indicates a significant difference in Xcn level between the wild type and the *ΔcpxR* mutant (*p* < 0.05, Student’s *t*‐test).

### 3.4 Roles of CpxR in xcn genes regulation

**Figure 1:**
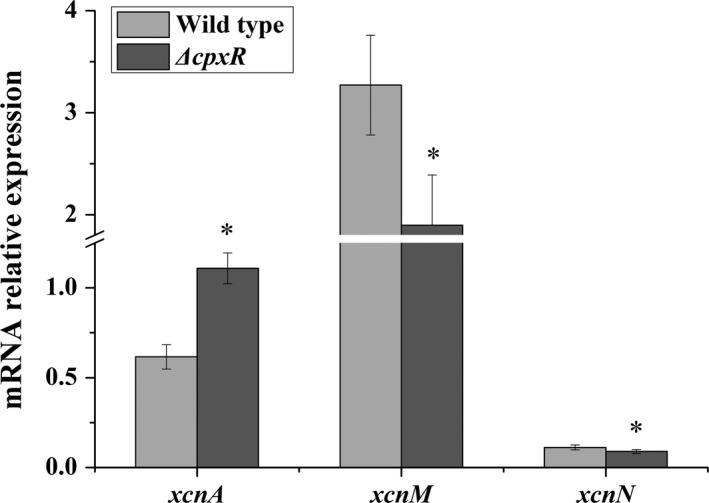
Expression levels of the main Xcn biosynthesis genes in the wild type and the *ΔcpxR* mutant. The transcript level of each gene was determined by qRT‐PCR. Total RNA was obtained from the wild type and *ΔcpxR* mutant at the exponential growth phase in TSB medium. Data are presented as the average ± *SD* for three replicates. An asterisk indicates a significant difference in a gene transcript level between the wild type and the *ΔcpxR* mutant (*p *<* *0.05, Student’s *t*‐test)

**Figure 2:**
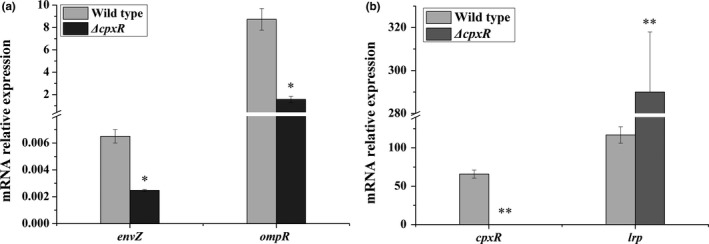
Expression levels of *envZ*,* ompR*, and *lrp* in the wild type and the *ΔcpxR* mutant. The transcript level of each gene was determined by qRT‐PCR. Total RNA was obtained from the wild type and *ΔcpxR* mutant of *X. nematophila* YL001 during exponential growth in TSB medium. Data are presented as the averages ± *SD* for three replicates. An asterisk indicates a significant difference in a gene transcript level between the wild type and the *ΔcpxR* mutant (*p *<* *0.05, Student’s *t*‐test). Double asterisks denote a significant difference at the 0.01 level

The biosynthetic gene cluster associated with the production of Xcns contains 14 genes (*xcnA‐xcnN*). To evaluate the functions of CpxR on *xcn* genes expression, we first determined the expression of the main genes required for Xcn biosynthesis by RT‐PCR. The levels of mRNA for *xcnA‐L* were increased while the expression of *xcnM* and *xcnN* was decreased in the *ΔcpxR* strain. Furthermore, the expression levels of three key genes (*xcnA*,* xcnM,* and *xcnN*) were verified by qRT‐PCR analysis (Figure 1). Compared with the wild‐type strain, the *ΔcpxR* strain exhibited a 1.80‐fold increase in the expression level of *xcnA*, while the expression levels of *xcnM* and *xcnN* were decreased by 0.42% and 0.20%, respectively. Meanwhile, in the *ΔcpxR* strain, the expression levels of *envZ* and *ompR* genes, encoding the protein of the OmpR/EnvZ two‐component system were decreased by 0.62% and 0.82%, respectively (Figure 2a). Besides, the transcript level of *lrp*, encoding the leucine‐responsive regulatory protein was increased 2.47‐fold relative to that of the wild strain (Figure 2b).

### 3.5 Antimicrobial activity of *Δ*cpxR mutant and wild‐type strain

Based on the finding of Herbert et al. (2007) that deleting *cpxR* resulted in a significant increase in antibacterial activity against *B. subtilis*, we determined the antibacterial activities of *ΔcpxR* mutant and wild‐type strain against five bacterial species. The cell‐free filtrate of the *ΔcpxR* mutant showed higher inhibitory effects than the wild type against *Bacillus cereus*,* B. subtilis*,* E. coli*, and *R. solanacearum* (Table 3). Especially, the antibacterial activity of *ΔcpxR* mutant against *B. subtilis* increased 33.32% relative to the parent strain.

**Table 3:** Inhibitory effect of the cell‐free filtrate of the wild type and the *ΔcpxR* mutant on five test bacteria

Bacteria	Inhibition zone diameter (mm)^a^	
	YL001	*ΔcpxR*
*Bacillus cereus*	26.56 ± 0.53	28.30 ± 0.58*
*Bacillus subtilis*	23.17 ± 0.63	30.89 ± 0.23*
*Staphylococcus aureus*	34.83 ± 0.61	35.17 ± 0.25
*Escherichai coli*	26.76 ± 0.48	29.37 ± 0.51*
*Ralstonia solanacearum*	23.06 ± 0.68	29.03 ± 0.85*

a Data are presented as the average ± *SD* for three replicates. An asterisk indicates a significant difference in the inhibitory effect between the wild type and the *ΔcpxR* mutant (*p* < 0.05, Student’s *t*‐test).

Furthermore, *ΔcpxR* strain also exhibited higher antimicrobial activities against 15 agricultural pathogenic fungi and oomycetes relative to the wild type (Table 4). Among the fungal and oomycete pathogens tested, the cell‐free filtrate of the *ΔcpxR* mutant showed inhibitory effects greater than 80% against *B. cinerea*,* P. capsici*, and *R. solani* with the inhibition rates increasing 20.31%, 19.54%, and 58.52% relative to the parent strain, respectively.

**Table 4:** Inhibitory effects of the cell‐free filtrate of the wild type and the *ΔcpxR* mutant on the mycelial growth of 15 plant pathogens

Pathogenic fungi	Inhibition rate (%)^a^	
	Wild type	*ΔcpxR*
*Botrytis cinerea*	75.02 ± 0.30	90.26 ± 0.67*
*Phytophthora capsici*	72.05 ± 0.18	86.13 ± 0.18*
*Rhizoctonia solani*	51.18 ± 1.42	81.13 ± 0.25*
*Exserohilum turcicum*	66.67 ± 0.94	72.84 ± 0.21*
*Physalospora piricola*	67.39 ± 1.68	70.48 ± 1.58
*Curvularia lunata*	56.70 ± 1.10	67.68 ± 1.22*
*Gaeumannomyces graminis*	56.13 ± 0.46	63.95 ± 0.43*
*Magnaporthe grisea*	36.02 ± 1.37	62.60 ± 1.12*
*Fusarium graminearum*	48.83 ± 1.79	56.37 ± 0.79*
*Verticillium dahliae*	34.09 ± 0.68	53.79 ± 1.02*
*Alternaria alternate*	40.30 ± 0.59	42.06 ± 0.86
*Fusarium oxysporum* f. sp. *cucumebrium*	35.71 ± 0.09	41.28 ± 0.38*
*Clomerela cinyulate*	19.39 ± 1.56	41.16 ± 0.96*
*Fusarium oxyporum* f. sp. *niveum*	28.83 ± 0.70	38.74 ± 0.92*
*Colletotrichum lagenarium*	20.53 ± 1.06	27.64 ± 0.73*

a The inhibitory rates of cell‐free filtrate of the wild type and the *ΔcpxR* mutant on the mycelial growth of the pathogens were tested after 1 week. Data are presented as the average ± *SD* for three replicates. An asterisk indicates a significant difference, the inhibitory effect between the wild type and the *ΔcpxR* mutant (*p* < 0.05, Student’s *t*‐test).

**Figure 3:**
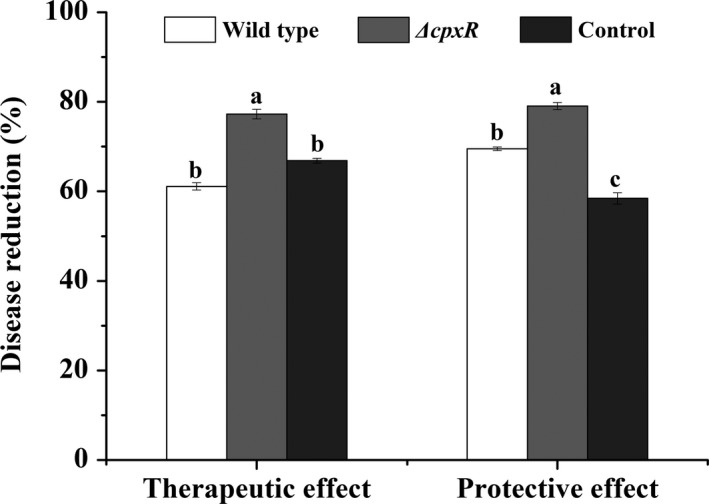
Effects of the cell‐free filtrates of the wild type and the *ΔcpxR* mutant on gray mold of tomato fruits caused by *Botrytis cinerea*. For both therapeutic and protective effects, the treatments were the cell‐free filtrates of the wild type and the *ΔcpxR* mutant and 1,000 times dilution of the 50% carbendazim was used as the positive control. Data are presented as the average ± *SD* for three replicates. Different lower case letters above the bars indicate significant differences at *p *<* *0.05

**Figure 4:**
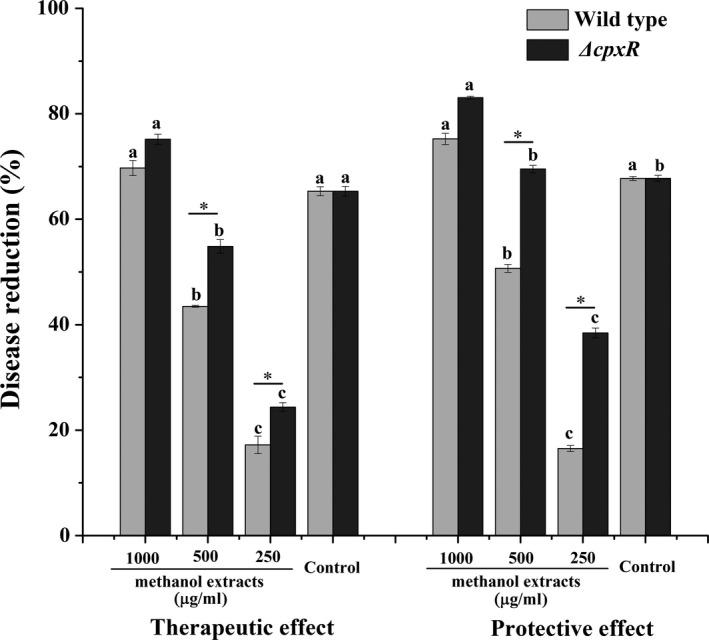
Effects of the methanol extracts of the cell‐free filtrate of the wild type and the *ΔcpxR* mutant on gray mold of tomato fruits caused by *Botrytis cinerea*. For both therapeutic and protective effects, the treatments are the methanol extract at 250, 500 and 1,000 μg/ml, and 1,000 times dilution of 50% Carbendazim was as chemical control. Data are presented as the average ± *SD* for three replicates. Different lower case letters above the bars indicate significant differences at *p *<* *0.05 between the methanol extract treatments with different concentrations and the control. An asterisk indicates a significant difference in the control efficacy between the wild type and the *ΔcpxR* mutant (*p *<* *0.05, Student’s *t*‐test)

Given the fact that *ΔcpxR* mutant had a significant inhibitory effect against *B. cinerea* of a 90.26% inhibition rate in vitro (Table 4), we determined the in vivo efficiency of *ΔcpxR* and wild‐type strain on tomato fruits infected with *B. cinerea*. The results showed that there was a significant efficiency of the cell‐free filtrates of *ΔcpxR* and wild‐type strain on detached tomato fruits infected with *B. cinerea*. The cell‐free filtrate of the *ΔcpxR* mutant strain exhibited higher therapeutic and protective effects than the wild type, and the therapeutic and protective effects increased 26.42% and 13.74% relative to the parent strain (Figure 3, Supporting Information Figure S6). There were significant effects (*p *<* *0.05) of the methanol extracts of *ΔcpxR* and wild‐type strain at 250, 500, and 1,000 μg/ml on detached tomato fruits infected with *B. cinerea* (Figure 4, Supporting Information Figure S6). The therapeutic and protective effects of *ΔcpxR* were higher than those of the wild type, and the protective efficacy was higher than the therapeutic efficacy at each treatment. At 250 and 500 μg/ml, both of the therapeutic and protective effects had significant differences between *ΔcpxR* and the wild type. At 1,000 μg/ml, the methanol extracts of *ΔcpxR* and wild‐type strain exhibited the therapeutic and protective effects greater than 70% but no significant differences were observed between *ΔcpxR* and the wild type (Figure. 4).

## 4 DISCUSSION

To elucidate the role of CpxR in the antibiotic production of *X. nematophila*, we construct a mutant strain of *cpxR* and determined the production of Xcns and the expression levels of *xcn* genes cluster (*xcnA‐M*) required for Xcn synthesis in *ΔcpxR* mutant. Also, the antimicrobial activities of the wild type and the *ΔcpxR* mutant were tested in vitro and in vivo.

Global regulators typically affect the production of small molecules in bacteria (Martınez‐Antonio & Collado‐Vides, 2003). Identification and manipulation of these global regulators could provide a powerful approach for discovery of new secondary metabolites and increase the production of useful molecules (Engel et al., 2017). As a response regulator, when the *cpxR* gene is deleted in *X. nematophila*, the level of Xcn1 in *ΔcpxR* was significantly increased compared to the wild type. Correspondingly, at the transcription level of *xcn* genes cluster (*xcnA‐M*), higher expression levels of *xcnA‐xcnL* were observed in *ΔcpxR* strain than those in the wild type while *xcnM* and *xcnN* were expressed at lower levels in *ΔcpxR* strain (Figure 1). As *xcnA‐xcnL* genes are responsible for the production of Xcn1 and other two genes, *xcnM* and *xcnN*, are responsible for the conversion of Xcn1 into Xcn2, the increased Xcn1 level in *ΔcpxR* may be the combined effects. These results may explain why the antimicrobial activity of *ΔcpxR* was increased in vitro and in vivo. Unexpectedly, Xcn2 in *ΔcpxR* strain was also increased, despite the low transcription levels of *xcnM* and *xcnN*. This may be related to the resistance mechanism utilized by the bacteria to avoid self‐toxicity (Park et al., 2009). In *ΔcpxR*, elevated Xcn1 levels might exceed a threshold for resistance, which stimulated the conversion of Xcn1 into Xcn2 based on *xcnM* and *xcnN* to maintain Xcn1 levels below a threshold of self‐toxicity. In spite of this, the level of Xcn1 was still enhanced in *ΔcpxR* mutant. Moreover, as described above, the cell density of *ΔcpxR* was higher than that of the wild type at the late stage of fermentation (Supporting Information Figure S4). These contradictory results seem to suggest that the deletion of *cpxR* can not only improve the production of Xcn1 but also increase the Xcn1 resistance of YL001. Similar phenomenon was also observed in the *ompR* mutant of *X. nematophila* (Park et al., 2009). As Xcn1 has greater potential in agricultural productions due to its excellent antibacterial activity (Mcinerney et al., 1991; Zhou et al., 2017), it is crucial to inhibit the conversion of Xcn1 into Xcn2. The addition of adsorber resin to the culture during the fermentation of *ΔcpxR* strain may be a practicable way to remove the excessive Xcn1 to reduce its conversion as well as cell toxicity (Gerth, Pradella, Perlova, Beyer, & Müller, 2003).

Xcn1 is a major antimicrobial compound of *X. nematophila* exhibiting a broad antimicrobial activity against *Alternaria alternata*,* Botrytis cinerea*,* Rhizoctonia solani,* and *Phytophthora* species (Huang et al., 2005, 2006; Zhou et al., 2017). Xcn2, however, shows substantially reduced bioactivities (Mcinerney et al., 1991; Yang et al., 2011; Zhou et al., 2017). Thus, the increased antimicrobial activity of *ΔcpxR* mainly depends on the elevated level of Xcn1. Besides, as other biosynthetic pathways of secondary metabolites may also be induced in the *ΔcpxR* strain, their contributions for antimicrobial activity need to be further explored.

Within *xcn* gene cluster (*xcnA‐M*), CpxR negatively regulates *xcnA‐L* but positively regulates *xcnMN* expression (Figure 1). As global regulator of transcription, CpxR binds to a specific promoter sequence upstream of their regulon and controls their expression. In *E*. *coli*,* cpxR* expression is autoregulated and CpxR binding site contains a consensus DNA sequence (5′‐GTAAA‐(N)_4‐8_‐GTAAA‐3′) (Yamamoto & Ishihama, 2006). In *X. nematophila*, the Cpx system is similar to that of the *E. coli* and the genetic structures of the *cpx* operon are similar in these two organisms (Herbert, Cowles, & Goodrich‐Blair, 2007). In this direction, we used the consensus DNA sequence (5′‐GTAAA‐(N)_4‐8_‐GTAAA‐3′) as a probe to scan the genome of *X. nematophila* ATCC 19061 by FIMO software to search for sequence similarities and CpxR binding sites upstream of *xcn* genes (Grant, Bailey, & Noble, 2011). Unfortunately, no strong CpxR consensus sequences were found, which may indicate that CpxR indirectly regulates *xcn* genes and other regulators are involved. Consistent with the discussion above, OmpR and Lrp, the global response regulators, also negatively and positively regulate the production of Xcn1, respectively (Engel et al., 2017; Park et al., 2009). As CpxR also positively regulates *ompR* and negatively controls *lrp* (Figure 2b), the likely regulatory hierarchy for Xcn production is one in which CpxR positively regulates OmpR, as well as negatively regulates Lrp, which in turn exerts certain effects on the expression of *xcn* biosynthetic clusters. However, members of the CpxR regulon that are either directly or indirectly regulated by CpxR remain to be distinguished.

## CONFLICT OF INTEREST

The authors have no conflicts of interest.
