# Supplementary material for: CpxR negatively regulates the production of xenocoumacin 1, a dihydroisocoumarin derivative produced by Xenorhabdus nematophila
Source: Microbiologyopen. 2018 Jun 11;8(2):e00674. doi: 10.1002/mbo3.674 (PMC6391269; doi:10.1002/mbo3.674)
Supplement: Supplementary file 1 [file MBO3-8-e00674-s001.docx]

**Supplementary Material**

**
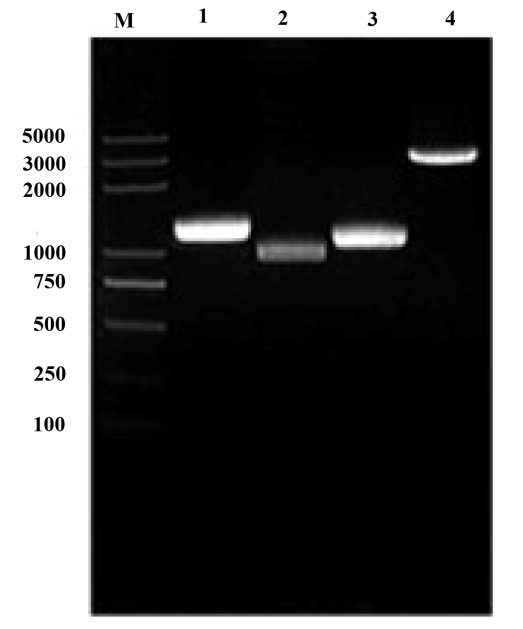
**

**Figure S1. Amplification and fusion of objective fragments.** Lane M, DNA molecular marker (Trans5k DNA marker); Lane 1, downstream flanking region; Lane 2, Km; Lane 3, upstream flanking region; Lane 4: products of fusion PCR.


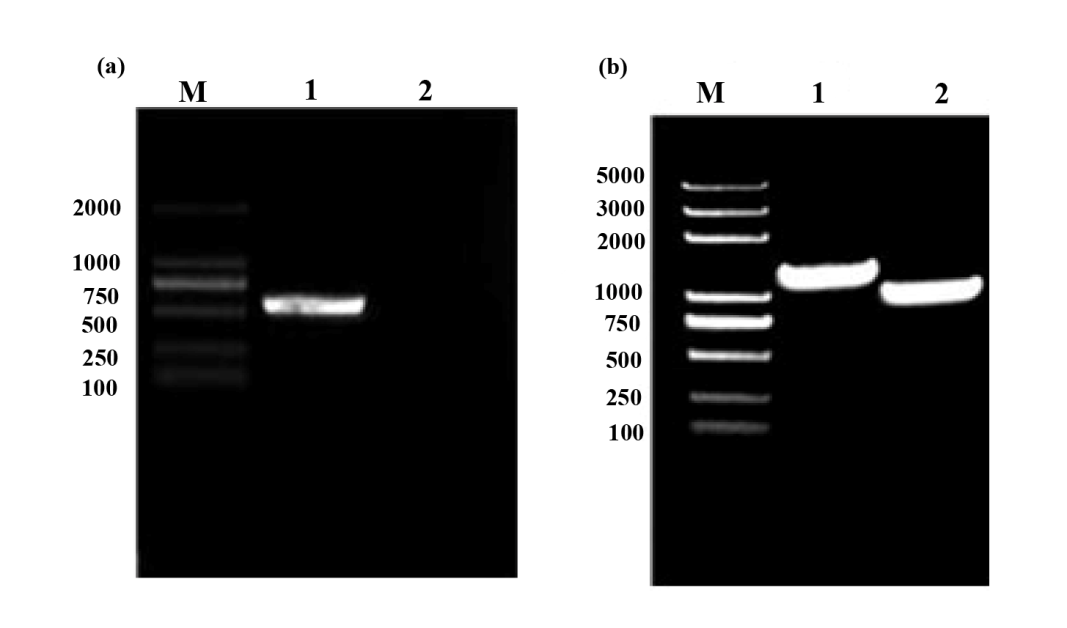


**Figure S2. The identification of the Δ*cpxR* mutant with internal and external primers.** (a) internal primers: Lane M, DNA molecular marker (Trans2k DNA marker); Lane 1, wild type; Lane 2, Δ*cpxR*; (b) external primers: Lane M, DNA molecular marker (Trans5k DNA marker); Lane 1, wild type 1; Lane 2, Δ*cpxR*.


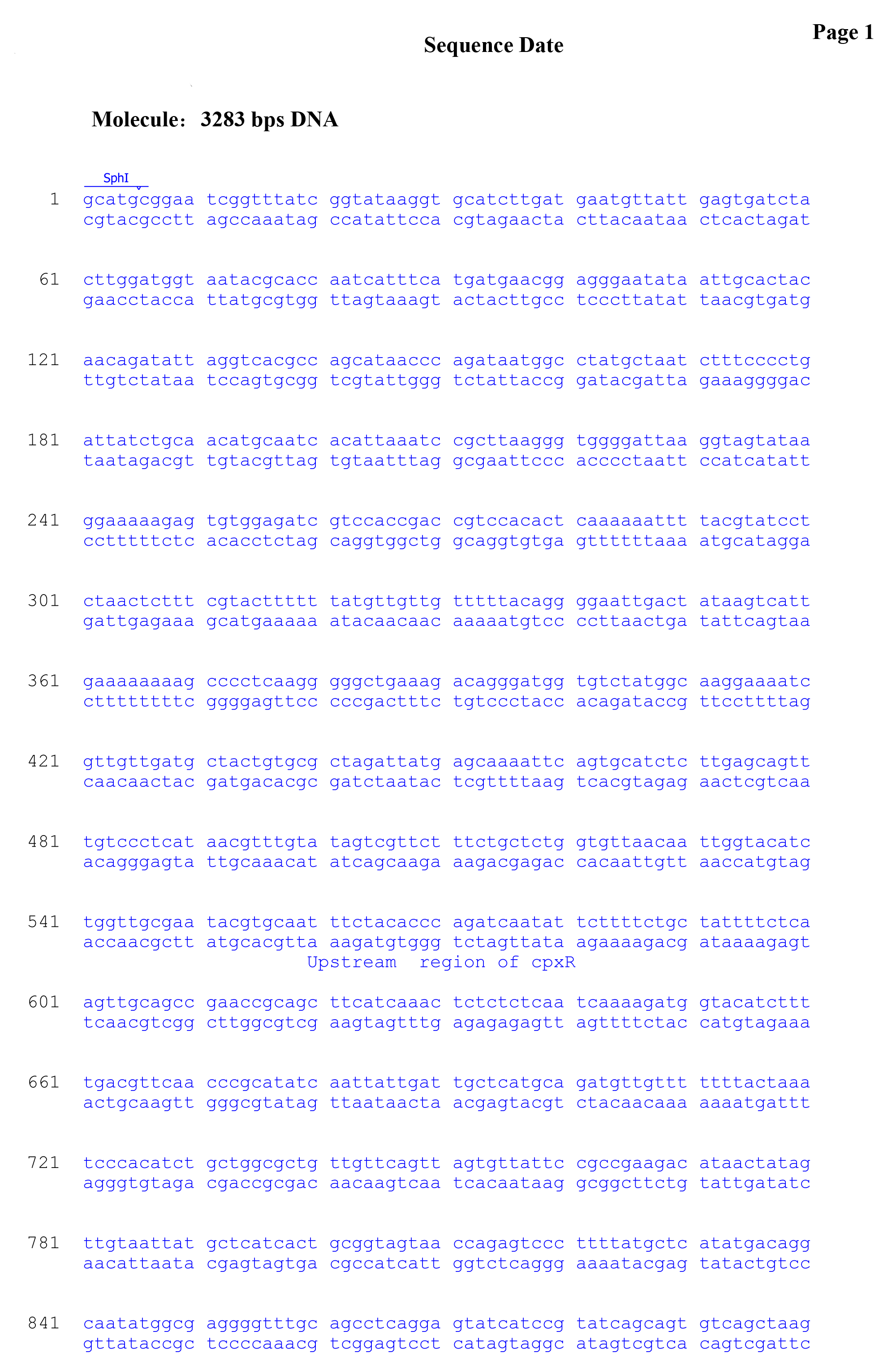


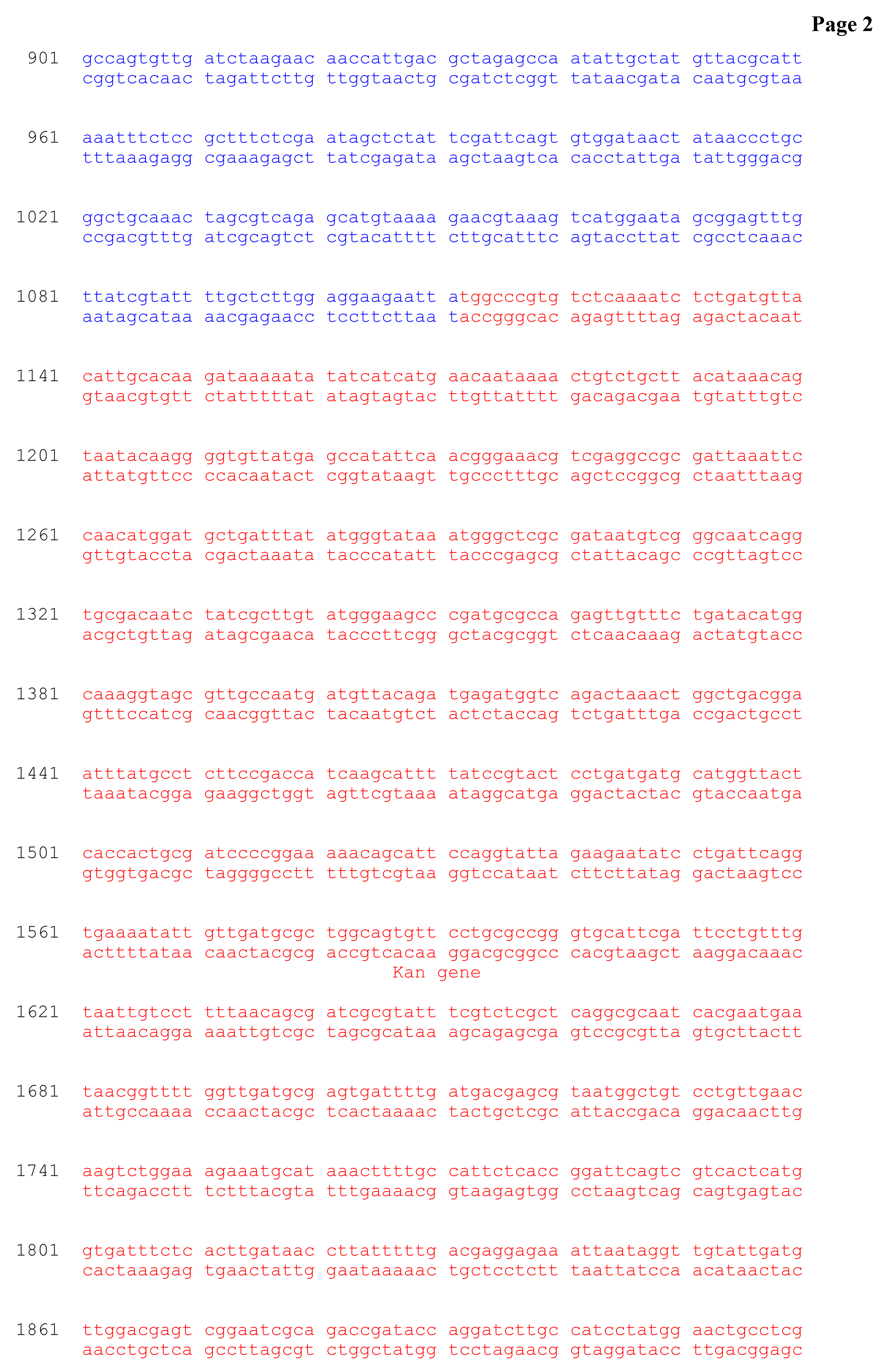


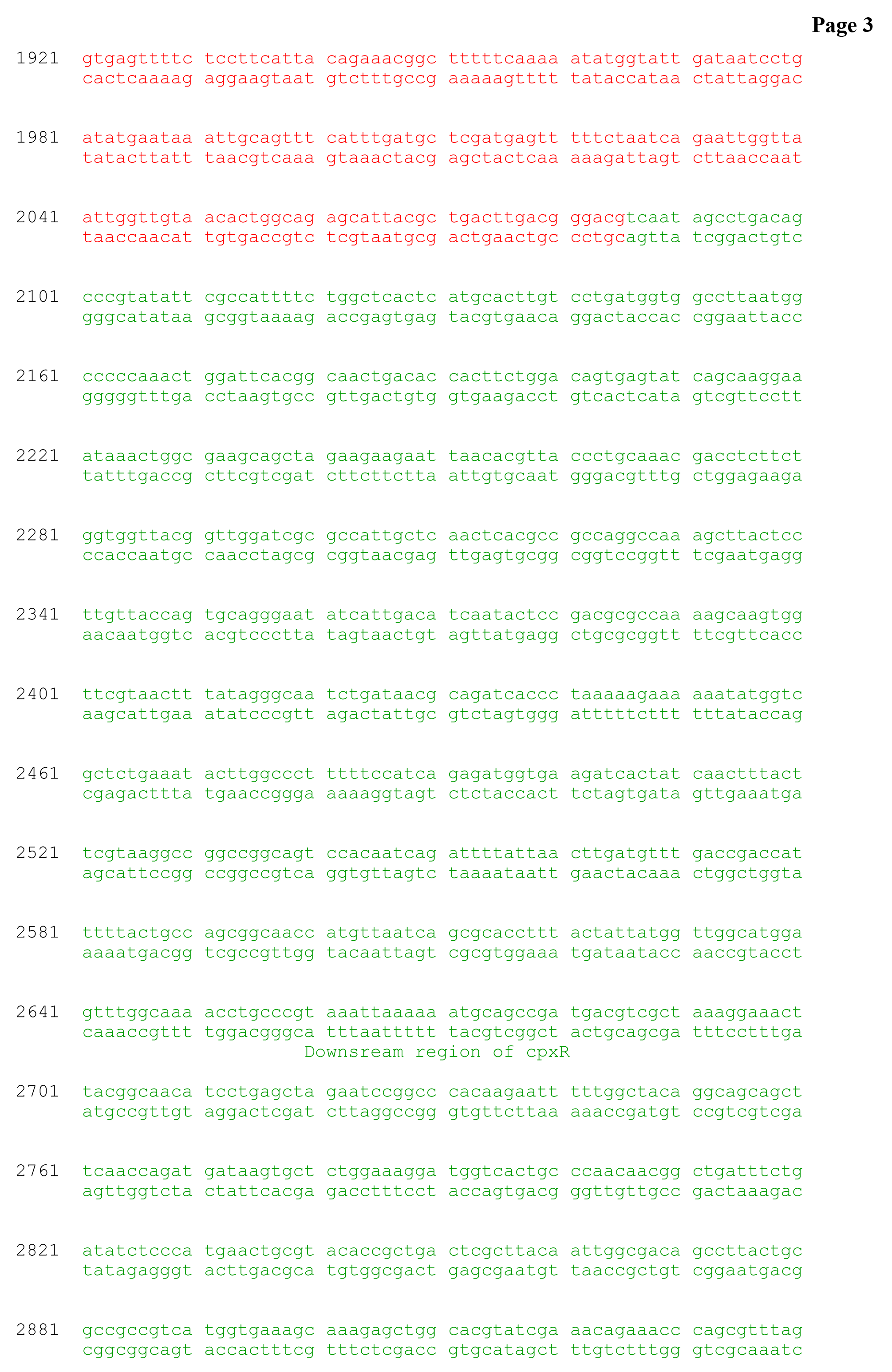


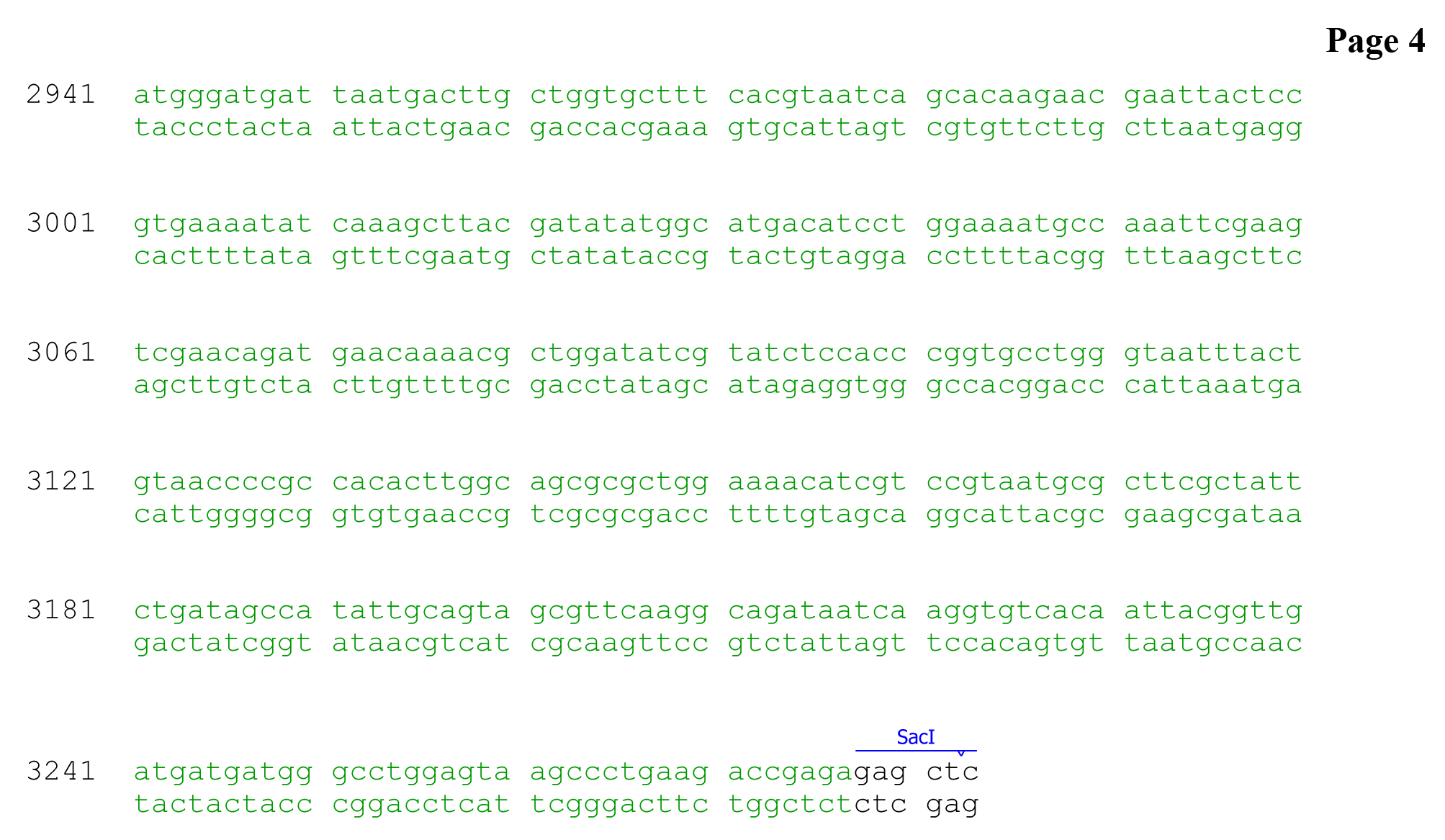


**Figure S3. Δ*cpxR* recombination region sequence.** The base pair in blue was the upstream of *cpxR*, the sequences in red was the *kan* gene of the screening tag copied from the plasmid pJCV53 with the antibiotic gene *kan*, the base pair in green was the downstream of *cpxR*.





**Figure S4** **The growth curves of the wild type and the Δ*cpxR* strain.** The cells were cultured in TSB medium and the growth was determined at different times by measuring the OD_600_. All experiments were performed in triplicate.

**
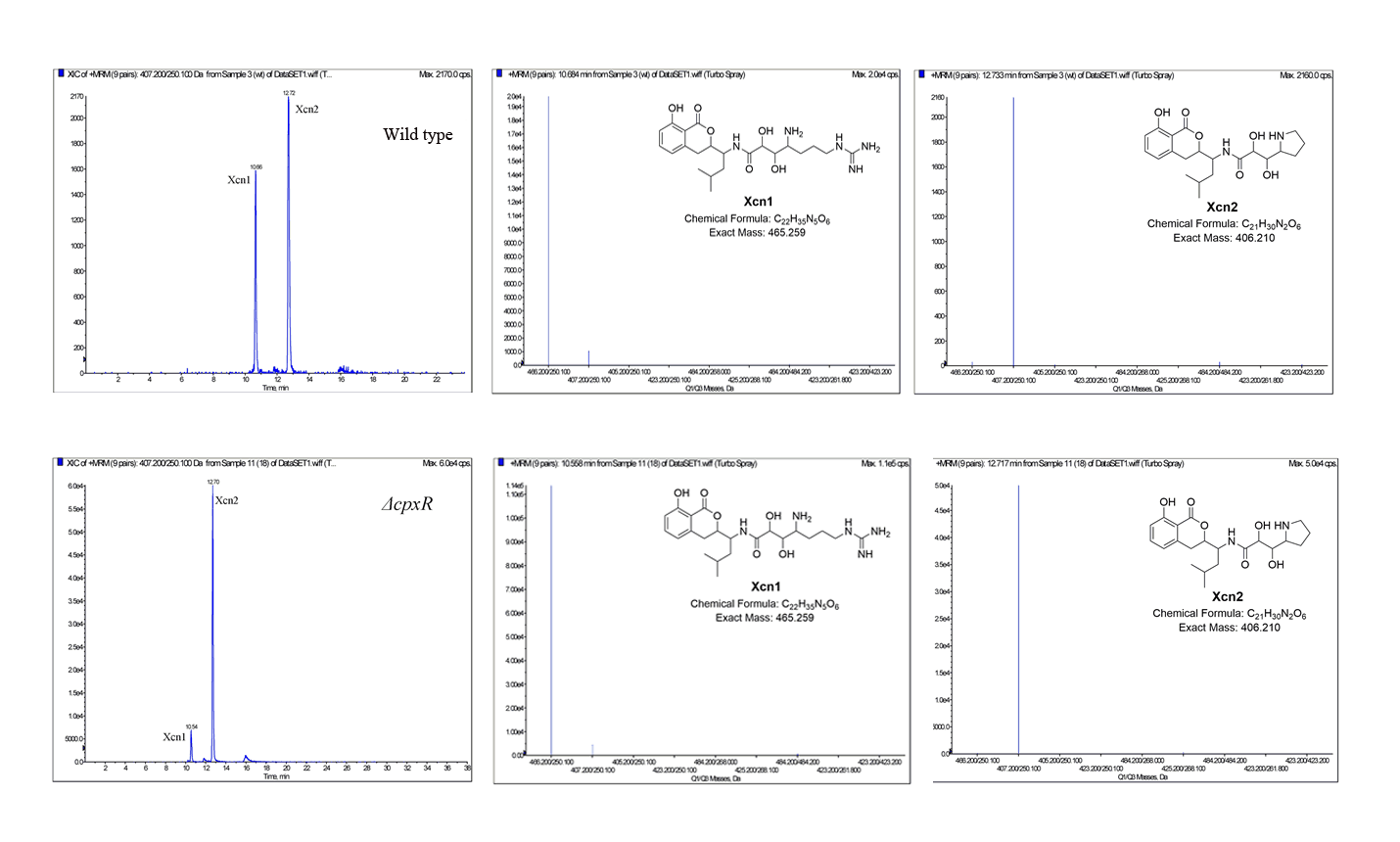
**

**Figure S5** **HPLC-MS analysis of Xcn1 and Xcn2 in the wild type and the Δ*cpxR*** **strain.** Up (wild type): HPLC chromatogram of the wild type (left), the molecular ion peak of Xcn1 ([M+H]^+^, 466 m/z)) (middle), the molecular ion peak of Xcn2 ([M+H]^+^, 407 m/z)) (right). Down (Δ*cpxR*): HPLC chromatogram of the Δ*cpxR* strain (left), the molecular ion peak of Xcn1 ([M+H]^+^, 466 m/z)) (middle), the molecular ion peak of Xcn2 ([M+H]^+^, 407 m/z)) (right).


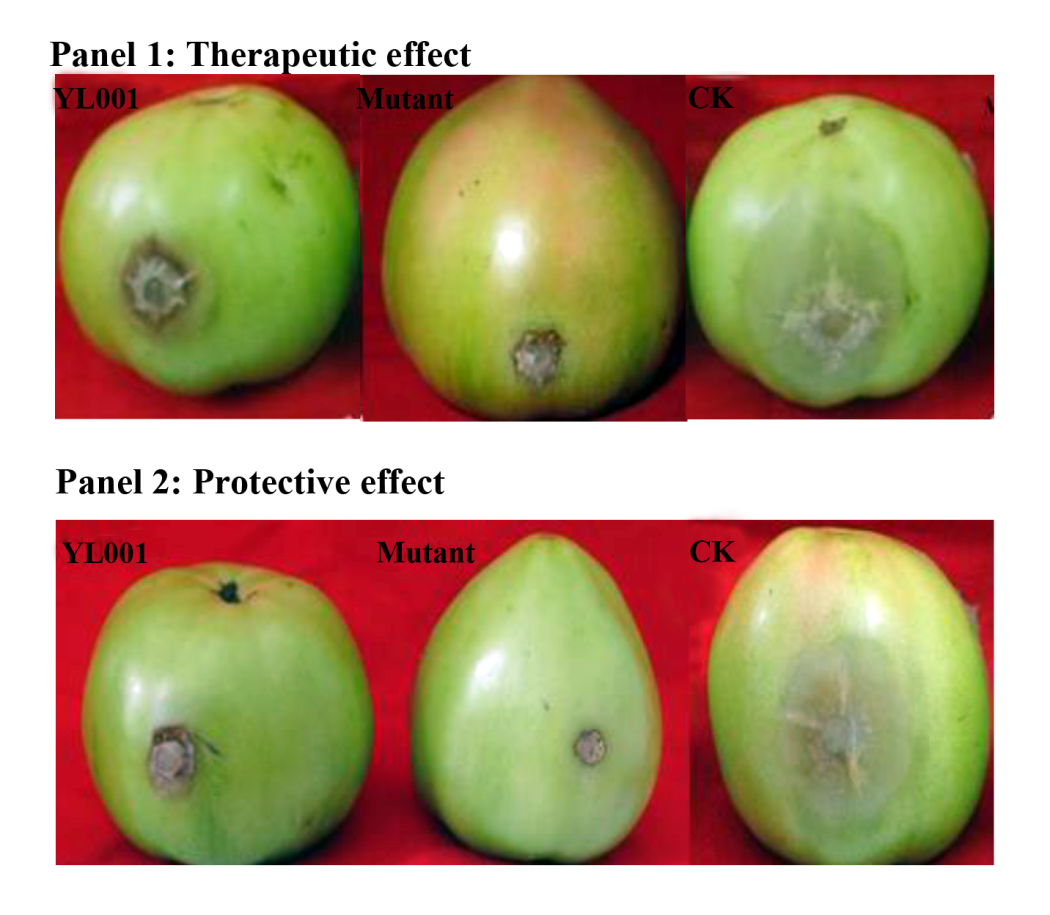


**Figure S6**. **The effects of the methanol extracts of the wild type and the Δ*cpxR*** **mutant on grey mold of detached tomato fruits caused by *Botrytis cinerea*.** Panel 1 (therapeutic effect): tomato fruits inoculated with agar discs from the edges of 4-day-old *B. cinerea* colony growing on PDA for 24 h prior to spray with (Left) methanol extract of wild type at 1000 μg /mL, (Middle) methanol extracte of Δ*cpxR* at 1000 μg/mL, and (Right) water control. Panel 2 (protective effect): tomato fruits inoculated with agar discs from the edges of 4-day-old *B. cinerea* colony growing on PDA for 24 h after spraying with (Left) methanol extract of the wild type at 1000 μg/mL, (Middle) methanol extract of the Δ*cpxR* mutant at 1000 μg/mL, and (Right) water control.

**Table S1.** Primers used in this study

| Primer | Sequence (5’→3’) ^a^ | Use |
| --- | --- | --- |
| *cpxR*-up-F | ACATGCATGCGGAATCGGTTTATCGGTATAAGGTGC | Mutant construction |
| *cpxR*-up-R | GAGACACGGGCCATAATTCTTCCTCCAAGAGCAAAATACGATAAC | Mutant construction |
| *Km*-F | GAGGAAGAATTATGGCCCGTGTCTCAAAATCTCT | Mutant construction |
| *Km*-R | CAGGCTATTGACGTCCCGTCAAGTCAGCG | Mutant construction |
| *cpxR*-down-F | TTGACGGGACGTCAATAGCCTGACAGCCCGTATATTCG | Mutant construction |
| *cpxR* -down-R | CCGAGCTCTCTCGGTCTTCAGGGCTTACTCC | Mutant construction |
| NB-F | AACCATTAAATATCCACGACCC | Mutant confirmation |
| NB-R | GATGGAAGGATTCAATGTCG | Mutant confirmation |
| WB-F | GAATAGCGGAGTTTGTTATCG | Mutant confirmation |
| WB-R | CACTGGTAACAAGGAGTAAGC | Mutant confirmation |
| *XcnA*-F | TTGAGACATTGGACACGGTGAAAG | qPCR analysis |
| *XcnA*-R | GCAGGTTGTTCCGTACTATCGAC | qPCR analysis |
| *XcnM*-F | GATTCGTATTGATGTTCATGGAGTTGATG | qPCR analysis |
| *XcnM*-R | ATGATCTTGAATATGCTCAGCCAACTG | qPCR analysis |
| *XcnN*-F | ATCATCTGGTGCATCACCTGTATC | qPCR analysis |
| *XcnN*-R | ATGGTTTCTTGATGAGGATGCTGAC | qPCR analysis |
| *cpxA*-F | TTCTGGACAGTGAGTATCAGC | qPCR analysis |
| *cpxA*-R | CACTGGTAACAAGGAGTAAGC | qPCR analysis |
| *cpxR*-F | CGCAGATGACTATCTCCCTAAACC | qPCR analysis |
| *cpxR*-R | CCGTCAAAACTGGCCTCC | qPCR analysis |
| *cpxP*-F | AGCGGATAACAATTTCACACAGGA | qPCR analysis |
| *cpxP*-R | CTCAAGTTGCAGCCGAACC | qPCR analysis |
| *envZ*-F | GCGGCTTCATCGTTGGCA | qPCR analysis |
| *envZ*-R | CGTATTGTGAGTGTTCCTGTC | qPCR analysis |
| *ompR*-F | GAGCAGGGATTTCAAGTTCGTAGTG | qPCR analysis |
| *ompR*-R | CTCAATCGGCAGACAGACAGT | qPCR analysis |
| *lrp*-F | TACGTTGAATCGTGGTGC | qPCR analysis |
| *lrp*-R | TAACAACATAAGTGCGGG | qPCR analysis |
| *recA*-F | TGATGAAGTTGTTGGTAGCGAAACG | qPCR analysis |
| *recA*-R | ACTCAGATCGATCAACTCTCCCAG | qPCR analysis |

^a^Restriction sites are denoted by the underlined sequences.

**Table S2.** Indicator bacteria used in this study

| Strains | characteristic (s) | |
| --- | --- | --- |
| *Bacillus cereus* | Gram-positive, facultative anaerobic spore-forming rod; |  |
| *Bacillus subtilis* | Gram-positive bacteria; |  |
| *Staphylococcus aureus* | Gram-positive bacteria, a kind of important human; pathogen, can be cause many serious infections; |  |
| *Escherichai coli* | Gram-negative, which could be cause severe diarrhea and sepsis; |  |
| *Ralstonia solanacearum* | Gram-negative, plant pathogenic bacterium; |  |
